# Aging-Related Accumulation of Truncated Oxidized Phospholipids Augments Infectious Lung Injury and Endothelial Dysfunction via Cluster of Differentiation 36-Dependent Mechanism

**DOI:** 10.3390/cells12151937

**Published:** 2023-07-26

**Authors:** Yunbo Ke, Pratap Karki, Yue Li, Kamoltip Promnares, Chen-Ou Zhang, Thomas L. Eggerman, Alexander V. Bocharov, Anna A. Birukova, Konstantin G. Birukov

**Affiliations:** 1 Department of Anesthesiology, University of Maryland School of Medicine, Baltimore, MD 21201, USA; 2 Division of Pulmonary and Critical Care Medicine, Department of Medicine, University of Maryland School of Medicine, Baltimore, MD 21201, USA; pkarki@som.umaryland.edu (P.K.);; 3 Department of Laboratory Medicine, Clinical Center, National Institutes of Health, Bethesda, MD 20894, USA; 4 National Institute of Diabetes, Digestive and Kidney Diseases, National Institutes of Health, Bethesda, MD 20892, USA

**Keywords:** aging, oxidized phospholipids, bacterial pathogens, lung injury, inflammation, CD36, short amphipathic helical peptides, L37pA

## Abstract

Truncated phospholipid oxidation products (Tr-OxPL) increase in blood circulation with aging; however, their role in the severity of vascular dysfunction and bacterial lung injury in aging groups remains poorly understood. We investigated the effects of six Tr-OxPL species: KOdiA-PC, POVPC, PONPC, PGPC, Paz-PC, and Lyso-PC on endothelial dysfunction and lung inflammation caused by heat-killed *Staphylococcus aureus* (HKSA) in young (aged 2–4 months) and old (aged 12–18 months) mice, organotypic culture of precisely cut lung slices, and endothelial cells (mLEC) isolated from young and old mice. HKSA and Tr-OxPL combination caused a higher degree of vascular leak, the accumulation of inflammatory cells and protein in bronchoalveolar lavage, and inflammatory gene expression in old mice lungs. HKSA caused a greater magnitude of inflammatory gene activation in cell and ex vivo cultures from old mice, which was further augmented by Tr-OxPLs. L37pA peptide targeting CD36 receptor attenuated Tr-OxPL-induced endothelial cell permeability in young and old mLEC and ameliorated KOdiA-PC-induced vascular leak and lung inflammation in vivo. Finally, CD36 knockout mice showed better resistance to KOdiA-PC-induced lung injury in both age groups. These results demonstrate the aging-dependent vulnerability of pulmonary vasculature to elevated Tr-OxPL, which exacerbates bacterial lung injury. CD36 inhibition is a promising therapeutic approach for improving pneumonia outcomes in aging population.

## 1. Introduction

Phospholipids are major building blocks of cell membranes and constituents of circulating lipoproteins. These lipid molecules undergo enzymatic oxidation via lipoxygenases and cyclogeneses or reactive oxygen species (ROS)-mediated nonenzymatic reactions, resulting in the generation of a wide variety of oxidized phospholipid (OxPL) products with profound biological activities [1,2]. Increased production and accumulation of OxPL is a pathological hallmark of several diseases, including atherosclerosis, lung inflammation, trauma, asthma, and acute respiratory distress syndrome [3,4,5,6,7]. More recent studies demonstrated the pathological role of *OxPL* in non-alcoholic steatohepatitis (NASH) in vivo [8,9]. From the perspective of lung function, the excessive oxidation of phospholipids becomes alarming when truncated OxPL (Tr-OxPL) species such as KOdiA-PC, POVPC, PONPC, PGPC, and Lyso-PC are produced at high levels that exhibit barrier disruptive and proinflammatory activities on pulmonary vascular endothelial cells (ECs) [10,11,12].

Elevated oxidative stress and inflammation are common features of aging, making the elderly population more vulnerable to various infections, including respiratory diseases [13,14]. For instance, the population over the age of 65 years accounts for over two-thirds of influenza-related hospitalizations and up to 90% of total yearly deaths in the United States [15,16]. Similarly, the incidence of severe sepsis is 20% higher in the elderly population, with 30–40% mortality compared to about 5% in younger patients [17,18]. The oxidative stress theory of aging states that an accumulation of reactive oxygen/nitrogen species with age causes oxidative damage to lipids, DNA, and proteins, leading to the functional loss and development of pathological conditions [14]. In turn, increased inflammation with aging, often described as ‘inflammageing’, arises due to immune dysregulation, cellular senescence, changes to microbiota composition, chronic infections, and oxidative stress-induced mitochondrial dysfunction [19]. Thus, this aging population exhibits an increased susceptibility to bacterial infections and ensuing prolonged inflammation. Interestingly, a number of recent studies, including from our own group, have reported higher levels of Tr-OxPLs in old mice lungs [12], plasma [20], and also in human blood plasma and serum [21,22]. However, the role of Tr-OxPLs in augmenting bacterial pathogen-induced lung injuries during aging remains to be investigated.

Cluster of differentiation 36 (CD36) is a class B scavenger receptor that is primarily involved in the uptake of OxPLs [23]. ECs have long been known to express CD36, and CD36 was first shown to bind OxLDL via the PC headgroup of OxPL by Boullier A et al. [24]. Studies have also shown that CD36 is the pivotal receptor activated by OxPLs during atherosclerosis [25] and by LPS during acute lung injury (ALI) [26]. In this regard, one of our recent studies has shown the protective effects of synthetic CD36 inhibitory peptides against LPS-induced ALI in vitro and in vivo. Yet, the role of CD36 in the modulation of Tr-OxPLs-induced endothelial dysfunction in relation to the aging process has not been examined. In the present study, we compared the individual and synergistic effects of Tr-OxPLs and *Staphylococcus aureus* in developing lung endothelial dysfunction in young and old mice. We also evaluated the protective effects of CD36 inhibitory peptide L37pA against Tr-OxPLs in vivo using mouse models of ALI and also in vitro using the cultures of precisely cut lung slices (PCLSs) and lung endothelial cells from both young and old mice. 

## 2. Materials and Methods

### 2.1. Chemicals

1-Palmitoyl-2-(5-oxovaleroyl)-*sn*-glycero-phosphocholine (POVPC), 1-palmitoyl-2-glutaroyl-*sn*-glycero-phosphocholine (PGPC), 1-palmitoyl-2-azelaoyl-sn-glycero-3-phosphocholine (Paz-PC), O-1-O-Palmitoyl-2-O-(5,8-dioxo-8-hydroxy-6-octenoyl)-L-glycero-3-phosphocholine (KOdiA-PC), 1-palmitoyl-2-(9-oxo-nonanoyl)-sn-glycero-3-phosphocholine (PONPC) and lysophosphocholine (Lyso-PC) were purchased from Cayman chemicals (Ann Arbor, MI, USA). L37pA was obtained from Pepmic Co. Ltd. (Suzhou, China). Heat-killed *Staphylococcus aureus* was purchased from InvivoGen (San Diego, CA, USA). Dulbecco’s Modified Eagle Medium (DMEM), fetal bovine serum (FBS), Dynabeads, type I collagenase, and penicillin/streptomycin were purchased from Thermo Fisher Scientific (Waltham, MA, USA). CD31 and ICAM-2 antibodies were from BD Biosciences (Franklin Lakes, NJ, USA) and VE-cadherin antibody was from Santa Cruz Biotechnology (Santa Cruz, CA, USA). Endothelial cell growth medium EGM-2 was obtained from Lonza (Allendale, NJ, USA). 

### 2.2. Animal Experiments

All the protocols involving animal care and treatments were approved by the University of Maryland Institutional Animal Care and Use Committee. Young (aged 2–4 months) and old (aged 18–24 months) C57/B6J mice from Jackson Laboratories (Bar Harbor, ME, USA) were used for the experiments. The breeders of CD36 knockout mice in C57B6 background were also purchased from Jackson Laboratories (Bar Harbor, ME, USA), and the offspring were used for experiments at ages 2–4 months and 18 months. HKSA (4 × 10^8^, bacterial particles) was injected intranasally, followed by the injection of the indicated Tr-OxPLs (10 mg/kg) into the jugular vein. L37pA (20 mg/kg, i.v.) was applied 30 min prior to Tr-OxPLs treatment where indicated. The control groups received the same volume of sterile saline. After 20–24 h, animals were sacrificed under ketamine (12 mg/kg, i.p.) anesthesia, and bronchoalveolar lavage (BAL) fluid was collected to analyze total cells and protein content as described earlier [23]. For Evans blue extravasation assay, the dye (30 mL/kg) was injected into the external jugular vein 2 h prior to the end of the experiment, and isolated lung images were captured as described previously [24]. Excised lungs were also used to isolate the total RNA to determine the mRNA expression of the selected endothelial inflammatory marker genes via qPCR.

### 2.3. Isolation and Culture of Mouse Lung Endothelial Cells

The isolation and culture of young and old mice lung ECs were carried out by following the procedures described earlier [27]. Briefly, the lungs harvested from freshly euthanized animals were kept on isolation media (DMEM supplemented with 20% FBS and 1% penicillin/streptomycin) and thoroughly minced with scissors. Lung homogenate was then digested with collagenase at 37 °C for 45 min with intermittent stirring. Next, tissue suspension was passed through a 5 mL syringe with a 20 G needle about 10–12 times and filtered through a 70 µM cell strainer. The cell suspension was centrifuged at 1200 rpm for 8 min at 4 °C, and cell pellets were dissolved in bead washing solution (PBS with 0.1% bovine serum albumin and 1% penicillin/streptomycin) and mixed with 25 µL of pre-prepared CD31-coated dynabeads. After robustly shaking at room temperature for 15 min, the cell suspension was washed five times with isolation media and resuspended in endothelial growth medium EGM-2 to seed in cell culture plates. After 24 h, the media was changed, and cells were allowed to grow until they reached 90% confluency. The cells were then detached using trypsin and the cell pellets were resuspended in bead washing solution followed by the addition of 20 µL of ICAM-2-coated dynabeads and incubation for 15 min with robust shaking. Finally, the cells were suspended in complete growth medium and seeded on the desired cell culture dishes for further experiments. The purity of the endothelial cells was routinely verified with VE-cadherin immunostaining, and only the isolation batch showing >90% VE-cadherin positive staining was used for experiments.

### 2.4. Precisely Cut Lung Slice Culture

PCLS isolation and culture was performed as described previously [28,29]. In brief, after euthanasia mouse lungs were inflated with 37 °C low-melting agarose (1.5% prepared with Hank’s Balanced Salt Solution). Inflated lungs were excised and immersed in ice-cold DMEM. Then, the lungs were sliced using a vibratome (Leica) by cutting transversely at 250 µM, and thus, the obtained uniform lung slices were placed on a 24-well cell culture plate (2–3 slices/well) and incubated at 37 °C for 2 h. After washing twice to remove excess agarose, PCLSs were cultured in DMEM.

### 2.5. Real-Time PCR

mRNA expression analysis was performed by carrying out quantitative real-time PCR on a Bio-Rad CFX96 with SYBR green. Briefly, after isolating the total RNA from the cultured cells or PCLS with the RNeasy plus kit (Qiagen, Hilden, Germany), cDNA was synthesized using the iScript cDNA synthesis kit (Bio-Rad, Hercules, CA, USA). Gene expression changes were determined by employing the ΔΔCt method after Ct values were normalized to GAPDH. The primers used for PCR are listed in Table S1.

### 2.6. Endothelial Permeability

An electric cell–substrate impedance sensing system (ECIS, Applied Biophysics, Troy, NY, USA) was used to measure endothelial permeability by monitoring transendothelial electrical resistance (TER) over time, as described previously [30]. Briefly, the cells seeded on the ECIS array plates with gold microelectrodes were subjected to the continuous measurement of TER, and normalized resistance was plotted against time to assess an increase in endothelial permeability.

### 2.7. Statistical Analysis

Data were presented as means ± S.D. For TER studies, six to eight independent measurements were performed for each condition. For qRT-PCR and Western blot analyses, three independent experiments were performed with each stimulation. The comparison between the control and the stimulated group was performed using unpaired Student’s *t* test and one-way ANOVA followed by post-hoc Fisher’s test, which was used for multiple groups. *p* < 0.05 was considered to be statistically significant.

## 3. Results

### 3.1. Old Mice Are More Susceptible to HKSA- and Tr-OxPL-Induced ALI

**Figure 1:**
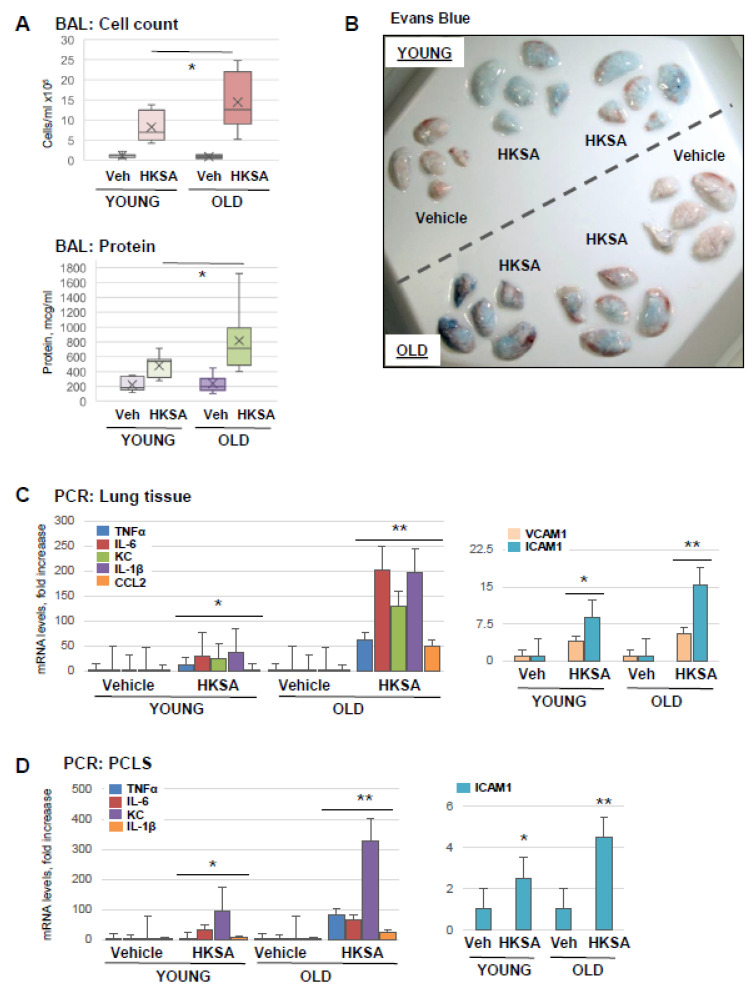
Old mice are more susceptible to HKSA-induced lung injury and inflammation. Young (aged 2–4 months) and old (aged 18–24 months) mice were exposed to HKSA (intranasal, 4 × 10^8^ bacterial particles) for 24 h. (**A**) BAL was collected from both groups, and total cell counts and protein content were determined; *n* = 6, * *p* < 0.05. (**B**) Evans blue dye was injected (i.v., 30 mg/kg) into mice 2 h before termination of the experiment, and excised lungs were visualized for the accumulation of the dye in lung parenchyma. Shown are representative images of six independent experiments. (**C**) Transcriptional activation of proinflammatory marker genes was measured by qRT-PCR analysis of total RNA extracted from lung tissue. Data shown as fold increase over control; *n* = 3, * *p* < 0.05 vs. Vehicle, ** *p* < 0.05 vs. young group. (**D**) PCLS were cultured as described in Methods and subjected to HKSA challenge (10^8^ cells/mL, 6 h). mRNA expression of indicated genes was determined via qRT-PCR *n* = 3, * *p* < 0.05 vs. Vehicle, ** *p* < 0.05 vs. young group.

Aging as a risk factor for ARDS severity is becoming increasingly recognized [31], and animal studies demonstrate the effects of aging as exacerbation factors in experimental models of ALI [12]. In this study, we first evaluated the magnitude of ALI caused by heat-killed *Staphylococcus aureus* (HKSA) and Tr-OxPLs in the young and old groups. Old mice (aged 18–24 months) developed more severe ALI in response to HKSA as compared to young (aged 2–4 months) counterparts, reflected by higher cell counts and protein content in BAL (Figure 1A). Evans blue extravasation assay further confirmed that HKSA induced more pronounced vascular leak in the old mice reflected by the greater accumulation of Evans blue dye in the lung parenchyma (Figure 1B). PCR analysis of lung tissue samples showed the increased expression of proinflammatory cytokines and EC adhesion molecules: TNFα, IL-6, KC, IL-1B, CCL2, VCAM-1, and ICAM-1 in the lungs of old mice (Figure 1C). Age-dependent augmenting effect on HKSA-induced inflammation was also reproduced in organotypic cultures of PCLS from old and young mice upon HKSA exposure ex vivo, as reflected by the enhanced transcriptional activation of TNFα, IL-6, KC, IL-1B, VCAM-1, and ICAM-1 (Figure 1D).

**Figure 2:**
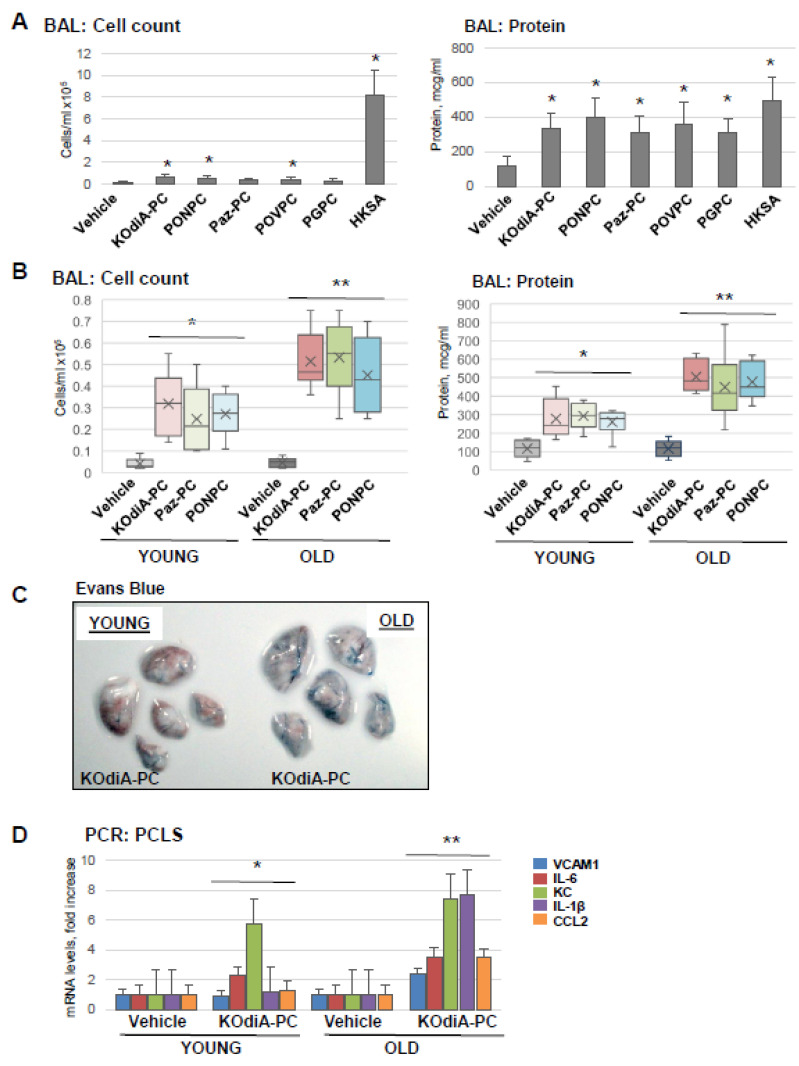
Tr-OxPLs induce more severe lung injuries in old mice. (**A**) Young mice were challenged with indicated Tr-OxPL species (i.v., 10 mg/kg, 24 h) followed by measurement of total cell and protein content in BAL; *n* = 6, * *p* < 0.05. (**B**–**D**) Young and old mice were exposed to KOdiA-PC or Paz-PC (10 mg/kg, 24 h). (**B**) Total cell and protein content in BAL; *n* = 6, * *p* < 0.05 vs. Vehicle, ** *p* < 0.05 vs. young group. (**C**) Evans blue extravasation assay to evaluate lung vascular leak. Shown are representative images of six independent experiments. (**D**) PCLS obtained from young and old mice were treated with KOdiA-PC in organotypic culture (40 µg/mL, 6 h), and qPCR was performed to measure the mRNA transcript levels of indicated proinflammatory genes; *n* = 3, * *p* < 0.05 vs. Vehicle, ** *p* < 0.05 vs. young group.

Next, we tested five Tr-OxPL species: KOdiA-PC, PONPC, Paz-PC, POVPC, and PGPC, for their capability to induce ALI in mice. The results showed that all five Tr-OxPLs caused the modest elevation of BAL cell counts compared to robust leukocyte recruitment to the lung caused by HKSA, suggesting the low proinflammatory potential of Tr-OxPLs per se. In contrast, Tr-OxPLs caused a pronounced increase in BAL protein content that was comparable with the effect of HKSA. These results suggest that increased lung vascular permeability is an early effect of acute Tr-OxPL elevation (Figure 2A). Comparative analysis showed that old mice exhibited higher BAL total cell counts and protein concentration upon challenge with KOdiA-PC or Paz-PC (Figure 2B). Likewise, old mice showed higher level of Evans blue accumulation in the lung parenchyma than in the young group, indicating enhanced vascular permeability in the old group (Figure 2C). PCLS isolated from lungs of young and old mice also showed that KOdiA-PC-induced increase in mRNA transcripts of VCAM-1, IL-6, IL-1B, and CCL2 was much higher in the old group (Figure 2D).

### 3.2. Tr-OxPLs Exacerbate HKSA-Induced Acute Lung Injury

**Figure 3:**
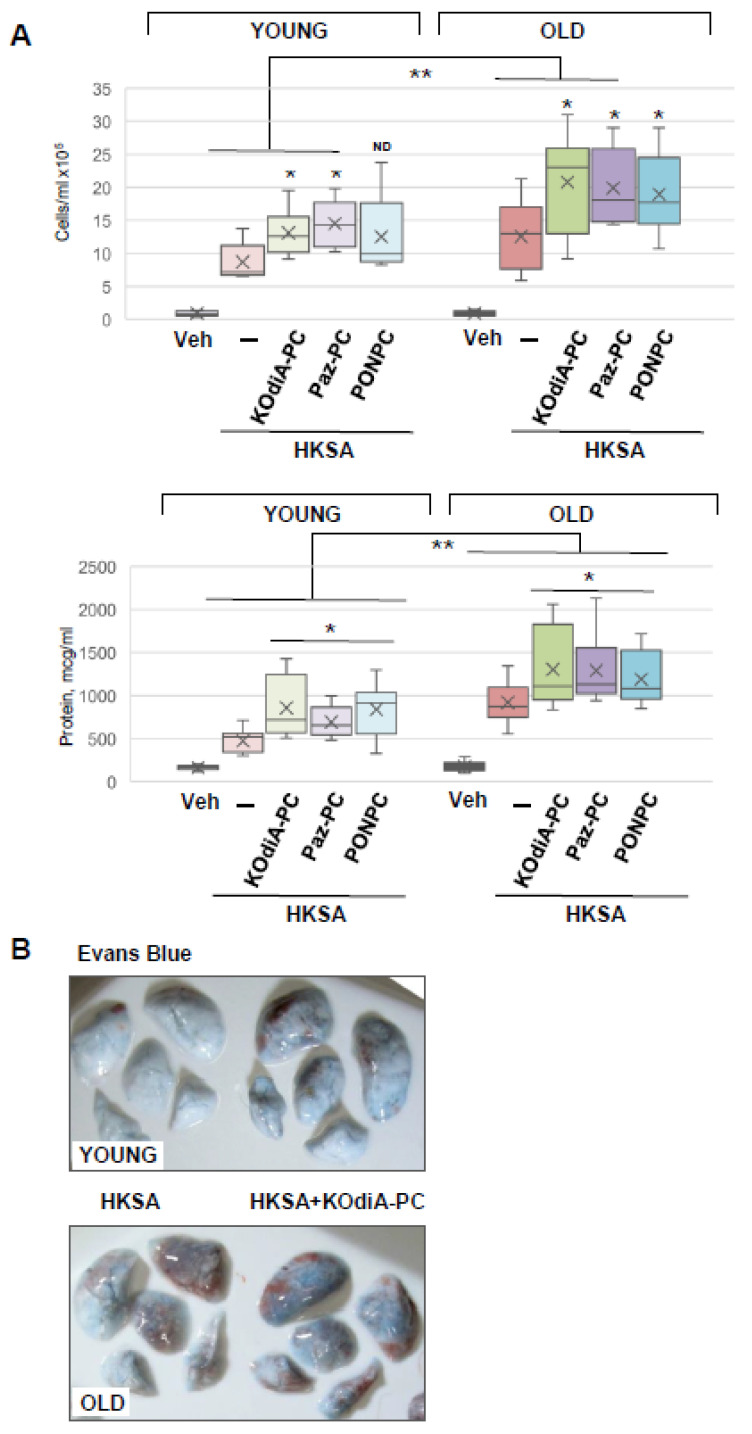
Tr-OxPLs exacerbate HKSA-induced lung injury in two-hit model of ALI. Young and old mice were co-treated with HKSA (intranasal, 4 × 10^8^ bacterial particles) and either KOdiA-PC, Paz-PC, or PONPC (10 mg/kg, i.v., 24 h). (**A**) BAL was collected to measure total cell counts and protein content; * *p* < 0.05 vs. HKSA alone, *n* = 6. In KOdiA-PC/HKSA model, BAL cell counts (13.11 +/− 3.67 vs. 20.78 +/− 7.78 cells × 10^5^, ** *p* < 0.04 Y vs. O) and protein concentration (857.39 +/− 354.39 vs. 1303.22 +/− 468.53 µg/mL, ** *p* < 0.02 Y vs. O) were compared between young and old groups. In Paz-PC/HKSA model, BAL cell counts (14.49 +/− 3.56 vs. 19.87 +/− 5.80 cells × 10^5^, ** *p* < 0.04 Y vs. O) and protein content (691.62 +/− 181.92 vs. 1291.49 +/− 435.79 µg/mL, ** *p* < 0.03 Y vs. O) were compared. In PONPC/HKSA model, BAL cell counts (12.53 +/− 6.38 vs. 18.97 +/− 6.36 cells × 10^5^, Y vs. O) and protein content (828.23 +/− 328.31 vs. 1189.52 +/− 318.95 µg/mL, ** *p* < 0.03 Y vs. O) were compared. ND, no difference. (**B**) Vascular leak was determined using Evans blue extravasation assay; representative lung images of three independent experiments are presented.

Since an increase in Tr-OxPLs levels and the risk of bacterial infections are commonly associated with aging, we next investigated the synergistic effects of these two stimuli and examined their age dependency. The results showed that although all three tested phospholipids, KOdiA-PC, Paz-PC, and PONPC, augmented HKSA-induced lung injury in both young and old mice, the net effect on lung dysfunction was more severe in the old group (Figure 3). In comparison to the young group, BAL cell count and protein content were significantly higher in the old mice treated with HKSA and KOdiA-PC or Paz-PC. In the young group treated with HKSA, PONPC induced moderate elevation in BAL cell count. While this effect was more pronounced in old mice, the difference between young and old groups did not reach statistical significance. In turn, protein content was increased in both groups, and it was significantly higher in BAL from the old animals compared to the young ones (Figure 3A). Evans blue extravasation assays confirmed that HKSA-induced lung vascular leak was worsened by KOdiA-PC treatment in both age groups, but more pronounced barrier dysfunction was observed in the old group (Figure 3B). More importantly, KOdiA-PC or Paz-PC-induced exacerbation of lung injury and inflammation was markedly higher in the old group than in younger counterparts.

### 3.3. HKSA and Tr-OxPLs Induce More Severe Inflammation in Endothelial Cell Isolated from Old Mice

**Figure 4:**
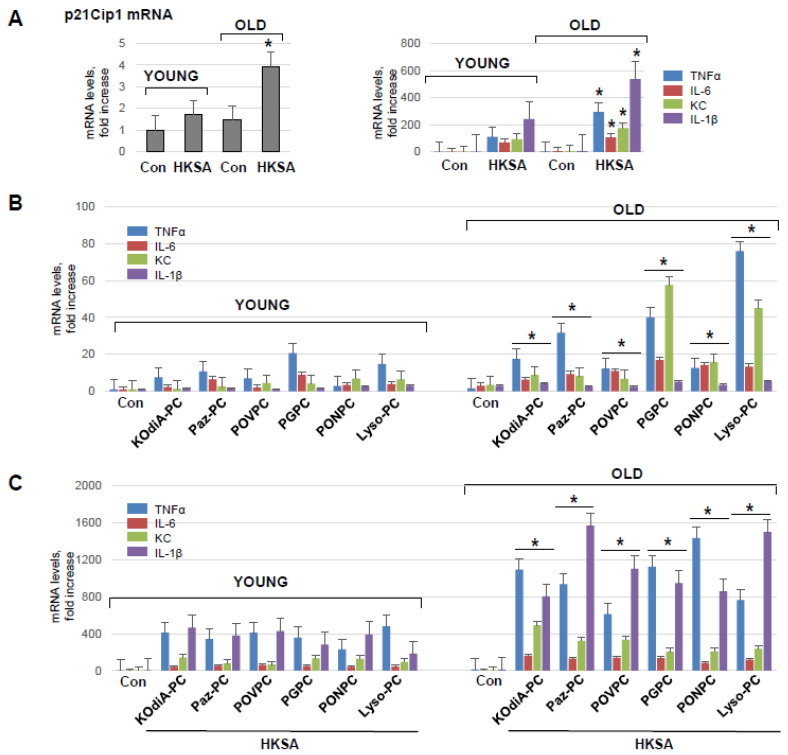
Lung ECs from old mice develop more pronounced inflammation in response to HKSA or Tr-OxPLs. (**A**) Isolated lung ECs from young and old mice were exposed to HKSA (10^8^ cells/mL, 6 h) followed by qPCR analysis of senescence marker p21^Cip−1^ and inflammatory markers VCAM-1, ICAM-1, IL-6, CCL2, TNF-α, KC, and IL-1β expression levels. Data presented as fold-increase over control; *n* = 3, * *p* < 0.05 vs. young group. (**B**) Lung ECs from young and old mice were exposed to indicated Tr-OxPLs (40 µg/mL), and mRNA transcript levels of selected endothelial inflammatory genes were determined by qPCR; *n* = 3, * *p* < 0.05 vs. young group. Data expressed as fold increase over control. (**C**) Both sets of ECs were subjected to HKSA (30 min), followed by the addition of indicated Tr-OxPLs (6 h). qPCR analysis of mRNA expression levels of indicated inflammatory genes; *n* = 3, * *p* < 0.05 vs. young group.

To investigate the direct effect of aging on pulmonary ECs’ functional response to injurious stimuli, we isolated primary lung ECs (mLECs) from young and old mice. mRNA expression of a senescence marker gene cyclin-dependent kinase inhibitor 1 p21^Cip−1^ was examined in both sets of mLEC at basal and HKSA-stimulated conditions. There was a significantly higher level of p21^Cip−1^ mRNA levels in mLEC from old mice as compared to those from young animals (Figure 4A, left panel). In addition, HKSA augmented the mRNA expression of p21^Cip−1^ in both young and old mLEC, but the increment was much higher in the old group. qRT-PCR analysis of young and old mLEC (Figure 4A, right panel) showed HKSA-induced expression of TNFα, IL-6, KC, and IL-1β transcripts, which was higher in mLEC from the old mice. In contrast to the age-dependent expression of these inflammatory markers, HKSA also caused a 20-fold induction of CCL2 and ICAM1 expression and a 10-fold induction of VCAM1 expression; however, there was no age-dependent difference in these parameters.

The incubation of mLEC with KOdiA-PC, PONPC, Paz-PC, POVPC, Lyso-PC, or PGPC also induced the transcriptional activation of TNFα, IL-6, KC, and IL-1β, although to a much lesser extent than HKSA (Figure 4B). Importantly, the magnitude of inflammatory response was significantly higher in mLEC from the old group. The two-hit model of mLEC challenged with Tr-OxPLs and HKSA (Figure 4C) showed augmented mRNA expression of all inflammatory markers upon combined HKSA and Tr-OxPL treatment as compared to challenge with either agonist alone (Figure 4A,B). Moreover, the magnitude of such response to HKSA and Tr-OxPL combination was further exacerbated by mLEC isolated from old mice (Figure 4C).

### 3.4. Lung ECs from Old Mice Are More Susceptible to Tr-OxPLs-Induced Barrier Dysfunction

**Figure 5:**
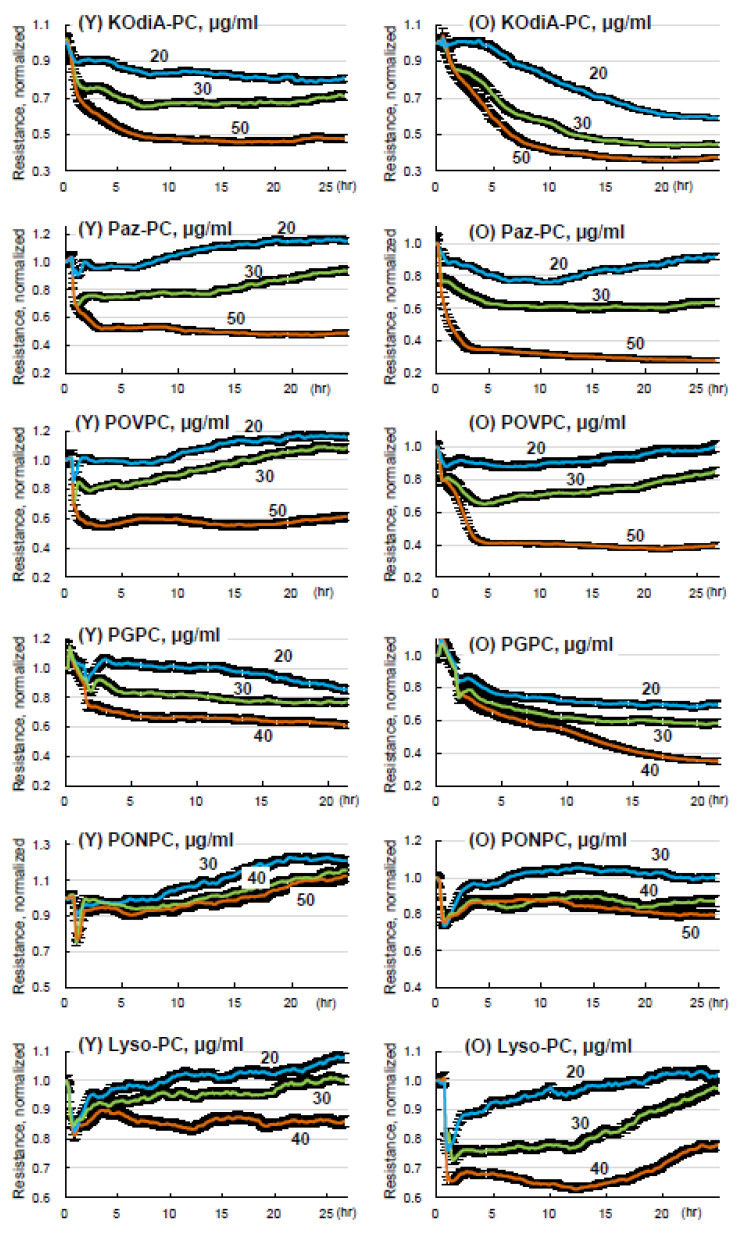
Tr-OxPLs induce more severe barrier disruption in lung ECs from old mice. ECs isolated from young and old mouse lungs were challenged with increasing concentrations (0–50 µg/mL) of indicated Tr-OxPLs, and TER was monitored for 25–30 h. Normalized resistance was plotted against time to determine endothelial permeability; *n* = 6. Comparative analysis of dose-dependent TER changes caused by each Tr-OxPL in lung ECs from young and old mice; *n* = 6.

We investigated whether the phenomenon of age-dependent exacerbation of inflammation and lung barrier dysfunction observed in animal models can also be detected in lung EC cultures isolated from young and old mice. Primary cultures of ECs from young and old lungs were exposed to increasing concentrations of KOdiA-PC, PONPC, Paz-PC, POVPC, Lyso-PC, and PGPC. A dose-dependent decline in TER reflecting increased permeability was observed in both young and old lung ECs. However, Tr-OxPLs-induced permeability response was more pronounced in lung ECs from the old group as compared to young EC counterparts (Figure 5). Of note, all tested Tr-OxPLs concentrations had no significant effects on cell viability, even at later time points up to 24 h. These observations are in agreement with our previous studies showing no changes in live/dead ratio in the cells treated with a high dose (100 µg/mL) of oxidized phospholipids mixture [32].

**Figure 6:**
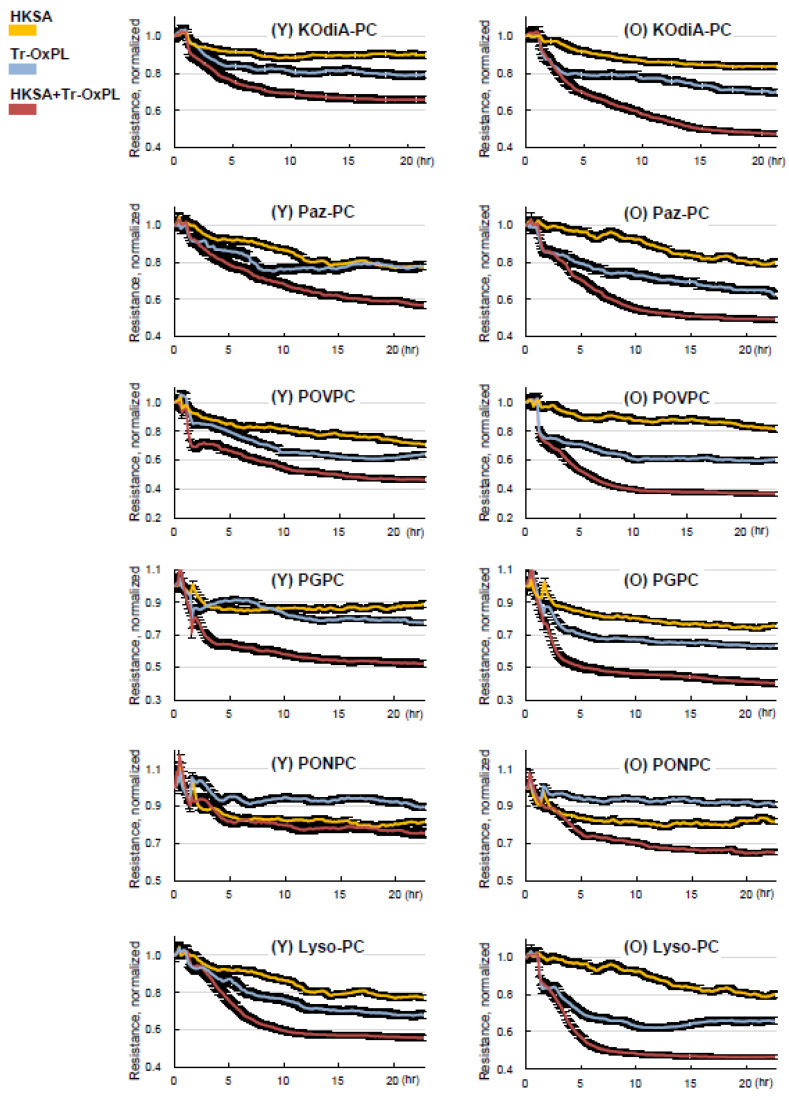
Tr-OxPLs cause more pronounced exacerbation of HKSA-induced barrier disruption in old mice lung EC. Lung ECs from young and old mice seeded on ECIS array plates were subjected to HKSA (30 min), followed by the addition of indicated Tr-OxPLs. TER was monitored, and normalized resistance was plotted against time. Comparative analysis of combined effects of HKSA and each Tr-OxPL on TER decline in lung ECs from young and old mice; *n* = 6.

We next investigated the effects of combined Tr-OxPL and HKSA treatment on permeability response by lung ECs isolated from young and old mice. Tr-OxPLs and HKSA exhibited synergistic effects on endothelial permeability in lung ECs from both age groups. However, the magnitude of barrier dysfunction caused by the combination of Tr-OxPLs and HKSA was much greater in ECs isolated from old mice (Figure 6). All six Tr-OxPL species demonstrated synergy with HKSA in increasing EC permeability, but this combination induced more profound barrier dysfunction in lung ECs from old mice.

### 3.5. Inhibition of CD36 Attenuates Tr-OxPLs-Induced ALI Both In Vitro and In Vivo

**Figure 7:**
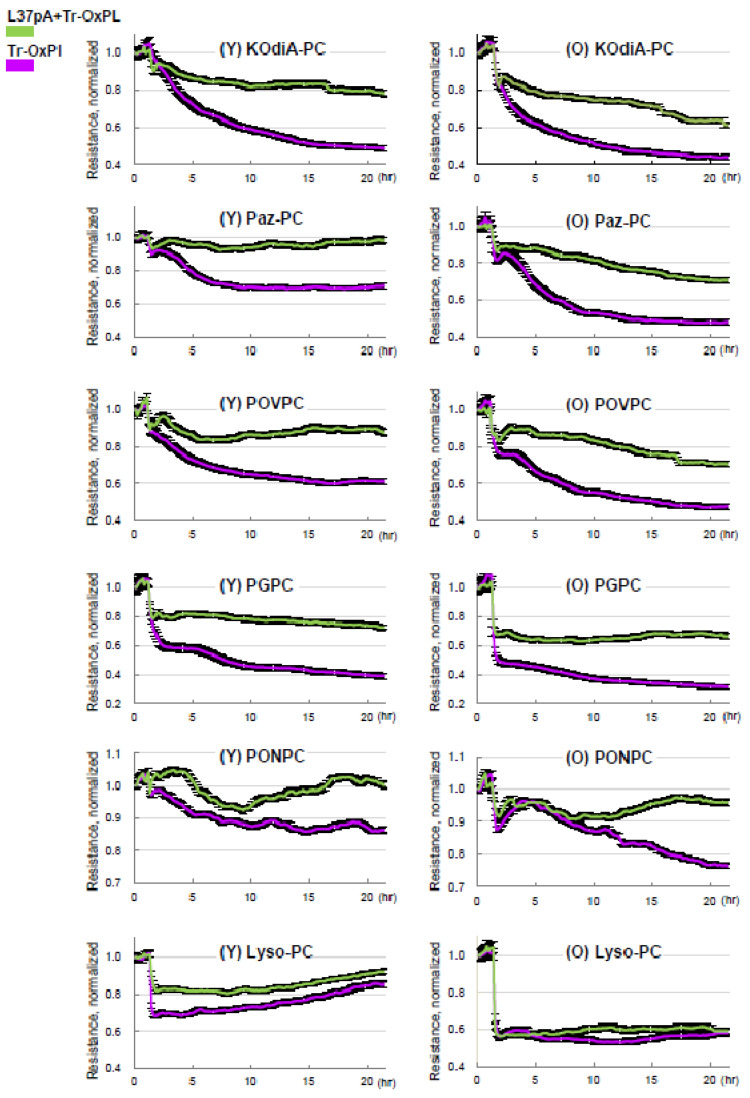
Inhibition of CD36 by synthetic peptide L37pA attenuates Tr-OxPLs-induced endothelial barrier dysfunction. Lung ECs from young and old mice were pre-treated with L37pA (25 µg/mL, 30 min) followed by the addition of indicated Tr-OxPLs, and TER was monitored over time. A decline in normalized resistance reflecting increased EC permeability is plotted against time. Effect of L37pA on TER decline in ECs from young and old mice caused by all Tr-OxPLs tested; *n* = 6.

Considering CD36 as the principal receptor interacting with Tr-OxPLs, we investigated whether the inhibition of CD36 offers protection against Tr-OxPL-mediated endothelial dysfunction. Published studies show that CD36 inhibitory peptides may rescue ALI caused by bacterial lipopolysaccharide [33]. Primary EC cultures isolated from mouse lungs were treated with CD36 inhibitory peptide L37pA prior to exposure to Tr-OxPLs. Measurements of TER demonstrated that L37pA attenuated barrier disruption caused by KOdiA-PC, Paz-PC, PONPC, POVPC, PGPC, or Lyso-PC in lung ECs from both young and old mice (Figure 7). The protective effect of L37pA was observed for all tested Tr-OxPLs except for Lyso-PC. These results suggest additional barrier-disruptive mechanisms activated by Lyso-PC.

**Figure 8:**
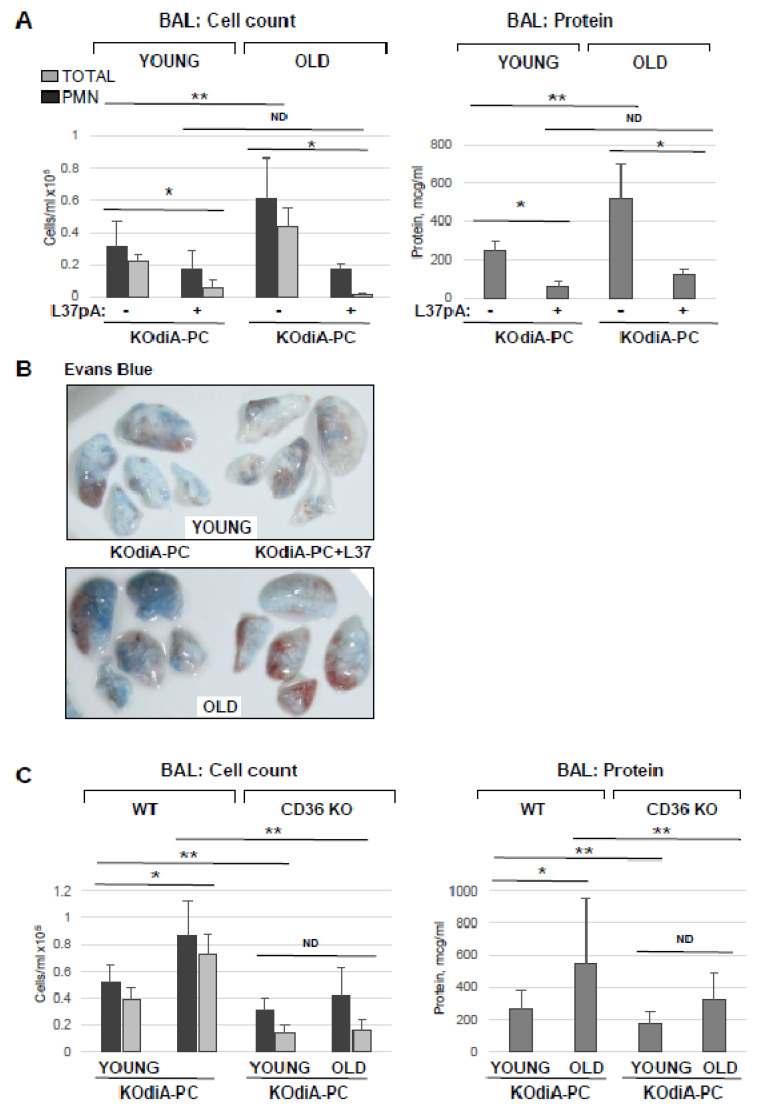
Inhibition of CD36 ameliorates Tr-OxPLs-induced lung injury in vivo. (**A**) Young and old mice received concurrent injection of L37pA (20 mg/kg, i.v.) and KOdiA-PC (10 mg/kg, i.v.). After 24 h, BAL was collected to determine total protein content, total cell counts and PMN counts; *n* = 6, * *p* < 0.05 vs. KOdiA-PC alone, ** *p* < 0.05 vs. young group. (**B**) Evans blue lung extravasation assay in KOdiA-PC-challenged mice. Shown are representative images of three independent experiments; *n* = 6 mice per condition. (**C**) Young and old CD36 knockout mice were exposed to KOdiA-PC; protein content, total cell and PMN counts were determined in BAL; *n* = 6, * *p* < 0.05 vs. young group; ** *p* < 0.05 vs. wild-type group; ND, no difference.

To investigate whether the protective effects of L37pA against Tr-OxPL-induced EC dysfunction can be recapitulated in vivo, we utilized KOdiA-PC as an example of a Tr-OxPL compound inducing lung dysfunction in young and old mice. L37pA (20 mg/kg) was injected intravenously 30 min following KOdiA-PC (10 mg/kg, i.v.) challenge. In agreement with the results described above, KOdiA-PC-treated old mice displayed elevated BAL total and PMN cell counts and protein content in comparison to the young group (Figure 8A). More importantly, L37pA abolished KOdiA-PC-induced increase in BAL protein content and strongly reduced total and neutrophil cell counts in BAL in both old and young mice. L37pA also strongly attenuated the KOdiA-PC-induced vascular leak in both groups of mice as determined by Evans blue dye lung extravasation assay (Figure 8B). The protective effects of L37pA were more pronounced in the old group. The direct role of CD36 in mediating Tr-OxPLs-induced ALI was further verified using a genetic model of CD36 knockout. The results showed that KOdiA-PC-treated CD36 knockout mice showed lower total and PMN cell counts as well as lower levels of protein in BAL samples, suggesting higher resistance to KOdiA-PC-induced lung dysfunction (Figure 8C). The trend to age-dependent elevation of BAL protein and cell counts was observed in a KOdiA-PC-challenged CD36 knockout group, but it did not reach statistical significance.

## 4. Discussion

An increase in the incidence, morbidity, and mortality of infectious respiratory diseases in older populations is well documented [16,17]. The age-dependent increase in oxidative stress and inflammation has been described by ‘oxidative stress theory’ and ‘inflammaging’, respectively, which have been proposed as two major phenomena responsible for higher age-related susceptibility to various diseases [13,14,19,34].

The present study demonstrates that aging directly contributes to the severity of ALI caused by bacterial infection and can be further exacerbated via the elevation of circulating Tr-OxPLs. The elevation of various Tr-OxPL species exhibited deleterious effects on lung vascular ECs in a dose-dependent manner. However, bacterial particles caused much more potent inflammatory activation and lung injury. Despite the high magnitude inflammation caused by HKSA, submaximal doses of Tr-OxPLs were able to further augment lung injury and EC dysfunction. These effects are dependent on CD36 signaling and can be controlled via the modulation of CD36 activity. Furthermore, this study identifies, for the first time, CD36 as a major receptor mediating the deleterious effects of Tr-OxPLs on endothelial function. We utilized a clinically relevant model of dose-controlled ALI caused by HKSA, which closely mimics the presence of killed bacteria after antibiotic treatment. Multiple studies have already established the potency of HKSA or heat-killed methicillin-resistant *Staphylococcus aureus* in inducing lung injury and inflammation [35,36,37].

Our results also demonstrate that Tr-OxPLs augmented EC dysfunction caused by Gram-positive bacterial particles and other inflammatory agonists. Similarly, Tr-OxPLs augmented HKSA-induced lung injury in vivo. Such synergy between circulating Tr-OxPLs and infectious agonists in the induction of lung EC inflammation and barrier dysfunction may be a cause of more severe bacterial lung inflammation in aging populations. These results also emphasize the role of pulmonary endothelium as a direct effector and target of plasma Tr-OxPLs. It is also important to note that “low” Tr-OxPL doses that did not cause EC barrier dysfunction alone, still augmented deleterious effects of HKSA. The synergistic effects of HKSA and Tr-OxPLs may have important clinical implications as they document that even the modest elevation of circulating Tr-OxPL may have significant adverse effects on the health of old individuals due to impaired immune function and increased susceptibility to bacterial infections. One can argue the physiological relevance of higher doses of Tr-OxPLs applied in this study but there are no published data on accurate levels of circulating OxPLs in human except the general consensus that these lipid mediators are elevated during various pathological states [21,38]. In justification of our model, PK/PD analysis of injected alkylated OxPLs analog (2–10 mg/kg) performed by our groups demonstrated micromolar concentrations (10–40 µM) of circulating OxPLs 30–60 min after injection (unpublished data). Moreover, the injected doses of Tr-OxPLs also should be interpreted cautiously given that these compounds undergo significant breakdown in circulation due to high activities of secreted phospholipases such as PLA2 in plasma. Additionally, even a significantly high dose of Tr-OxPLs (100 µg/mL) did not cause any adverse cytotoxic effects on cultured ECs [32].

Our published studies demonstrated elevated production of Tr-OxPLs in aging mice challenged with LPS [12], suggesting dysregulated antioxidant defense in comparison to young counterparts, which further contributed to the aging-dependent exacerbation of LPS-induced lung injury. The novel findings of this study investigating responses of lung ECs isolated from adult and old mice demonstrate that ECs from old mice acquire additional vulnerability to Tr-OxPLs. Such phenomenon of age-dependent cellular vulnerability has been described in the literature. For example, the aging of ECs has been shown to enhance their sensitivity to apoptotic and proinflammatory stimuli such as OxLDL and TNFα; this effect was in part due to senescence-associated eNOS downregulation leading to the upregulation of caspase-3 activity [39]. Another emerging mechanism of cellular aging, so-called stress-induced premature senescence (SIPS) mechanism [40], is activated by stressful environments: radiation, infection, chemical agents, etc. The results of this study suggest that Tr-OxPLs and HKSA may play a role in such a stressor-promoting SIPS mechanism, which becomes further accelerated in ECs isolated from old mice exposed to Tr-OxPLs. Such amplification of the aging process at the molecular level may augment cellular response to a broad range of pathologic insults.

Our results show that old ECs retained this feature even during a few passages in culture. In addition, experiments with PCLS from adult and old mice that were challenged with HKSA in organotypic culture demonstrate similar age-dependent augmentation of HKSA-induced inflammation and accelerated senescence reflected by the higher induction of senescence markers by HKSA in PCLS and ECs isolated from old mice. Interestingly, the augmenting effects of various tested Tr-OxPLs in young and old animals were not uniform, with some species such as Paz-PC, KOdiA-PC showing stronger variations, while POVPC and PGPC showing a mild increase, and a minimal difference was observed with Lyso-PC. The varying potency of Tr-OxPLs in exacerbating bacteria-induced lung injury warrants further investigation aimed at the development of more selective therapies to mitigate the effects of the most harmful Tr-OxPLs.

In order to investigate the cellular mechanisms of Tr-OxPL-induced EC dysfunction in ALI models, we focused on CD36, one of the principal receptors that directly targets oxidized LDL and phospholipids. Surprisingly, the potential role that CD36 has in the modulation of Tr-OxPLs-induced regulation of endothelial function has not been explored so far. To fill this gap in knowledge, we utilized CD36 inhibitory synthetic peptide L37pA to examine its protective effect against Tr-OxPLs-induced augmentation of ALI. Our earlier study showed that chemically synthesized amphipathic helical peptides targeting CD36 rescued lung injury and inflammation caused by Gram-negative endotoxin LPS [33]. In addition to LPS, *S. aureus* particles can be recognized by CD36 in addition to canonical TLR2/6 receptor mechanism. This interaction likely occurs via the recognition of lipoteichoic acid present on the bacterial wall, which contains phospholipid moieties. In addition, CD36 expressed by macrophages regulates phagocytosis. These factors: CD36 expression by other inflammatory cells and possible interaction of CD36 both with Tr-OxPLs and *S. aureus* add a complexity to the interpretation of animal experiments, which demonstrated the attenuation of Tr-OxPL/HKSA-induced endothelial dysfunction and ALI via the pharmacological and molecular inhibition of CD36.

Our new data show the strong attenuation of endothelial barrier dysfunction by L37pA caused by several Tr-OxPLs, except for Lyso-PC, where only a marginal protective effect was observed (Figure 7). More strikingly, L37pA was effective against Tr-OxPL- and HKSA-induced dysfunction of ECs isolated from both young and old mice, highlighting the potential of this peptide in rescuing more severe lung injury caused by elevated Tr-OxPLs in older age groups. The protective effects of L37pA were successfully recapitulated in the in vivo model of ALI, where the inhibitor demonstrated high efficacy in both young and old mice. Another strong piece of evidence supporting an essential role of CD36 during Tr-OxPLs-induced ALI was obtained from experiments with young and old CD36 knockout mice. The results showed the better resistance of CD36 knockout mice to KOdiA-PC-induced vascular leak and inflammation compared to wild-type matched controls.

While the results presented in this study firmly support CD36 as a critical receptor mediating the deleterious effects of Tr-OxPLs on lung endothelium, one important question remains unanswered: how do CD36 inhibitors protect against Tr-OxPLs-induced endothelial dysfunction? Possible scenarios include the blocking of Tr-OxPLs internalization by L37pA, thus minimizing the bioavailability and toxic effects of the lipid products, additional anti-inflammatory activities of the inhibitor via interactions with other receptor(s), and interference with barrier-disruptive signaling cascades activated by Tr-OxPLs. These mechanisms warrant further investigations.

In conclusion, this study demonstrated, for the first time, that the deleterious effects of elevated Tr-OxPL associated with aging affect the functional stability of lung ECs and exacerbate ongoing infectious lung injury. These age-dependent complications may be alleviated by L37-pA, a synthetic peptide antagonizing CD36 function. Such protective effect was more pronounced in old animals. These results, together with data showing less severe Tr-OxPL-induced lung dysfunction in CD36 KO mice, further support the CD36-dependent mechanism of L37-pA-mediated lung protection in aging mice. Collectively, our findings suggest that synthetic peptides targeting CD36 hold strong therapeutic potential in mitigating aging-associated endothelial dysfunction and improving lung inflammatory syndromes in aging populations.

## Data Availability

Not applicable.

## Supplementary Materials

The following supporting information can be downloaded at: https://www.mdpi.com/article/10.3390/cells12151937/s1, Table S1: List of primer sequences used for RT-PCR.
