# Supplementary material for: Aging-Related Accumulation of Truncated Oxidized Phospholipids Augments Infectious Lung Injury and Endothelial Dysfunction via Cluster of Differentiation 36-Dependent Mechanism
Source: Cells. 2023 Jul 26;12(15):1937. doi: 10.3390/cells12151937 (PMC10416939; doi:10.3390/cells12151937)
Supplement: Supplementary file 1 [file cells-12-01937-s001.zip › cells-2356367-supplementary.pdf]

Table S1. List of primer sequences used for RT-PCR

Gene name	Forward Primer	Reverse Primer
Mouse TNF-α	CTGTAGCCCACGTCGTAGC	TTGAGATCCATGCCGTTG
Mouse IL-6	CCGGAGAGGAGACTTCACAG	TCCACGATTTCCCAGAGAAC
Mouse IL-1β	GAAATGCCACCTTTTGACAGTG	TGGATGCTCTCAGGACAG
Mouse CCL2	GTTGGCTCAGCCAGATGCA	AGCCTACTCATTGGGATCATCTTG
Mouse GAPDH	AATGTGTCCGTCGTGGATCT	AGACAACCTGGTCCTCAGTG
